# A CT-based radiomics model for preoperative risk stratification of gastrointestinal stromal tumors

**DOI:** 10.3389/fonc.2026.1671745

**Published:** 2026-02-19

**Authors:** Tianyi Wan, Yanping Song, Xiaolian Wang, Pei Huang, Zonghuo Wang, Bing Fan, Wentao Dong

**Affiliations:** 1 Department of Radiology, Jiangxi Provincial People’s Hospital, The First Affiliated Hospital of Nanchang Medical College, Nanchang, China; 2 Department of Quality Control, The First Affiliated Hospital, Jiangxi Medical College, Nanchang University, Nanchang, China; 3 Medical College of Nanchang University, Nanchang University, Nanchang, China

**Keywords:** contrast-enhanced CT, gastrointestinal stromal tumors, machine learning, radiomics, risk stratification

## Abstract

**Background:**

Gastrointestinal stromal tumors (GISTs) are common mesenchymal tumors with variable malignancy potential, making accurate preoperative risk stratification crucial for treatment planning. Traditional methods rely on pathological and clinical features but often overlook tumor heterogeneity. This study aims to develop and validate a CT-based radiomics model for GISTs risk stratification to improve clinical decision-making.

**Methods:**

This retrospective, multi-center study developed and validated a radiomics-based risk prediction model in accordance with the TRIPOD-ML statement. It included 123 patients with GISTs from two hospitals, divided into training (n=68), testing (n=30), and external validation (n=25) cohorts. Tumor delineation was performed using 3D segmentation on venous-phase contrast-enhanced CT scans. Radiomics features (n=1784) were extracted and refined using feature selection methods, including LASSO and ANOVA. Six machine learning algorithms were evaluated, and the support vector machine (SVM) model demonstrated optimal performance. Model evaluation included metrics such as AUC, calibration curves, and decision curve analysis (DCA).

**Results:**

The SVM-based radiomics model achieved robust performance, with AUC values of 0.906 (95% CI: 0.812–0.964) in the testing cohort and 0.867 (95% CI: 0.724–0.956) in the external validation cohort. Calibration curves indicated strong agreement between predicted and observed outcomes, while DCA highlighted significant clinical utility across different thresholds. Key radiomics features provided accurate differentiation between Lower Risk and Elevated Risk groups, aligning with clinical stratification needs.

**Conclusions:**

The developed CT-based radiomics model offers a reliable, externally validated tool for GISTs risk stratification, addressing limitations of traditional methods by incorporating tumor heterogeneity and enhancing predictive accuracy. This model has the potential to guide personalized treatment strategies, particularly in distinguishing patients with GISTs requiring adjuvant therapy from those suitable for surgical resection alone. This study was approved by the appropriate ethics committee with a waiver of informed consent.

## 1 Introduction

Gastrointestinal stromal tumors (GISTs) originate from the interstitial cells of Cajal, the pacemaker cells of the gastrointestinal tract (1). GISTs are the most common type of mesenchymal tumors in the digestive system, primarily occurring in the stomach and small intestine, and accounting for approximately 1 to 2% of all malignant digestive tract tumors (2). These tumors exhibit diverse biological behaviors and are generally considered potentially malignant due to their unpredictable progression (3).

Key prognostic factors for GISTs include tumor location, size, mitotic rate, and tumor rupture. Based on these factors, Joensuu et al. (4) proposed a modified version of the National Institutes of Health (NIH) risk stratification system, categorizing GISTs into four levels: very low, low, intermediate, and high risk. This classification has been widely adopted in clinical practice to predict recurrence risk and guide treatment decisions (5). However, despite its widespread use, the NIH risk stratification system has certain limitations. It mainly relies on clinical and pathological features, which may not fully capture the tumor’s biological behavior (6, 7). Additionally, it does not consider tumor heterogeneity or microstructural details, which are important for accurate prognosis (8). Radiomics, by extracting high-dimensional features from medical images, provides a more detailed and comprehensive assessment of tumors, addressing these shortcomings (9–11). By incorporating imaging data, radiomics may offer a more precise and reliable tool for GISTs risk stratification, ultimately improving treatment planning and clinical decision-making.

While prior studies have applied radiomics to GISTs risk stratification, many of these have relied on single-center data and have not validated their models using independent external datasets, limiting their generalizability (12–14). To address these limitations, this study aims to utilize data from two hospitals for developing a CT radiomics-based model for GISTs risk stratification, classifying tumors into two groups: Lower Risk (combining very low and low-risk categories) and Elevated Risk (combining intermediate and high-risk categories), which is validated with external datasets. The treatment approaches for these two groups differ significantly. For patients in the Lower Risk group, treatment typically involves surgical resection, as the recurrence risk is relatively low, and adjuvant therapy is often not required (15, 16). In contrast, patients in the Elevated Risk group are more likely to benefit from additional therapeutic interventions, such as adjuvant imatinib therapy, due to the higher recurrence risk and the potential for metastatic spread (17, 18). Therefore, this model aims to offer a more accurate and reliable predictive tool to assist in the stratified treatment of GISTs in clinical practice.

## 2 Methods

Reporting Guideline: This study was reported in accordance with the Transparent Reporting of a Multivariable Prediction Model for Individual Prognosis or Diagnosis–Machine Learning (TRIPOD-ML) statement. A completed TRIPOD-ML checklist is provided as **Supplementary Material**.

### 2.1 Patient cohort

This retrospective study was approved by the local Institutional Review Board (IRB) (Jiangxi Provincial People’s Hospital), and informed written consent was waived by the IRB. All methods were performed in accordance with the relevant guidelines and regulations.

This study included 123 patients with GISTs from two hospitals between January 2020 and August 2024. The 98 cases from Jiangxi Provincial People’s Hospital were randomly divided into a training cohort (n=68) and a testing cohort (n=30) using stratified sampling by the binary NIH risk category (Lower vs. Elevated Risk), with a fixed random seed (seed=42) to ensure reproducibility. The 25 cases from The First Affiliated Hospital of Nanchang University constituted the independent external validation cohort. The inclusion criteria were: (1) histopathologically confirmed diagnosis of GISTs; (2) preoperative enhanced CT scan performed within 15 days prior to surgery; (3) clear risk stratification based on pathological findings. The exclusion criteria were: (1) prior neoadjuvant treatment with imatinib or other tyrosine kinase inhibitors before CT; (2) lack of preoperative contrast-enhanced CT or poor image quality (e.g., artifacts).

Patients were categorized based on the NIH risk stratification system into two groups: Lower Risk (combining very low- and low-risk categories) and Elevated Risk (combining intermediate- and high-risk categories).

### 2.2 Clinical and imaging data collection

Baseline clinicopathological characteristics, including age, gender, gastrointestinal bleeding (GI-bleed), and abdominal pain, were extracted from patient medical records. Tumor-related features such as shape, boundary, location, cystic degeneration, diameter, and calcification were obtained from the imaging PACS system. Statistical analyses, including two-sample t-tests and χ² tests, were conducted using SPSS 24.0 software (IBM) to compare these variables between the training and testing groups. No missing data were present in the collected clinical, conventional imaging for the 123 included patients. Additionally, to assess the independent predictive value of these clinical and conventional imaging features for NIH risk stratification, a multivariable logistic regression analysis was performed using data from all 123 patients. The binary NIH risk category (Lower vs. Elevated) served as the dependent variable. Odds ratios (ORs) with 95% confidence intervals (CIs) were calculated.

### 2.3 Imaging examination methods

All patients were instructed to fast for a minimum of 6 hours and to drink 600–1000 mL of water prior to undergoing contrast-enhanced CT examinations. The CT scan encompassed the entire abdominal region. Detailed CT imaging protocols are provided in **Table 1**.

**Table 1:** CT imaging protocols.

CT scanner	256-slice Brilliance iCT (Philips Healthcare, Cleveland, Ohio, USA)	128-slice OPTIMA 660 (GE healthcare Japan Corporation, Hino-Shi, Toyo, Japan)
Tube voltage (kV)	120	120
Tube current (mAs)	220	100-200, Smart mAs
Rotation time (s)	0.75	0.4
Detector collimation (mm)	128×0.625	64×0.625
Field of view (mm)	360×360	360×360, Large body
Matrix	512×512	512×512
Reconstruction Section thickness (mm)	1.0	1.25
Contrast agent type	Iomeron, Bracco	Iomeron, Bracco
Contrast agent concentration	400 mg I/mL	400 mg I/mL
Contrast agent dosage	1.125 mL/kg body weight	1.125 mL/kg body weight
Contrast agent infused rate	3.0 mL/s	3.0 mL/s
Venous phase interval time	70 s after injection of contrast agent	70 s after injection of contrast agent

### 2.4 ROI segmentation and feature extraction

A radiologist (T.Y.W) with over 5 years of experience in oncology imaging performed manual ROI segmentation for GISTs on contrast-enhanced CT images in the venous phase. The SHUKUN platform (https://medresearch.shukun.net/project) was utilized for precise ROI delineation, employing its image labeling and algorithm tools. DICOM-format CT images were uploaded to the platform for visualization, adjustment, scaling, and 3D reconstruction. ROIs were manually contoured on each slice using 3D drawing tools within the labeling software. Subsequently, the Fit Boundary tool, based on a seed region-growing algorithm, was applied to refine the segmentation of bright and dark ROIs within the manually defined boundaries. Each ROI was labeled as either Lower Risk or Elevated Risk. After completing the labeling, the system automatically extracted radiomics features, which were saved to a database. Radiomics feature extraction was performed using PyRadiomics in SHUKUN platform, following principles and guidelines from its documentation (https://pyradiomics.readthedocs.io/en/latest/customization.html). To ensure stability, a robust extraction method was applied, addressing challenges in capturing surrounding features and achieving reliable results. A senior radiologist (B.F), with 15 years of experience, reviewed and validated the contours, with disagreements resolved through collaborative discussion to reach consensus.

To further evaluate segmentation reproducibility, an inter− and intra−observer agreement analysis was conducted on 30 randomly selected cases. A second radiologist (X.L.W., with 8 years of experience in abdominal imaging) independently segmented all tumors, and the primary radiologist (T.Y.W.) re−segmented the same cases after a 48−hour interval with image order randomized. Dice similarity coefficient (DSC) and intraclass correlation coefficient (ICC, two−way mixed−effects model) were used to quantify spatial overlap and feature−level reproducibility, respectively.

### 2.5 Feature selection for radiomics data

After ROI segmentation, prior to feature extraction, all CT images were resampled to an isotropic voxel size of 1.0 × 1.0 × 1.0 mm³ using linear interpolation to standardize spatial resolution across patients and scanners. The PyRadiomics package in the SHUKUN platform extracted a comprehensive set of 1784 quantitative radiomics features. These features encompassed shape, texture, and intensity measurements, including first-order statistics, shape descriptors, and various texture features derived from the Gray-Level Co-occurrence Matrix (GLCM), Gray-Level Dependence Matrix (GLDM), Gray-Level Run-Length Matrix (GLRLM), Gray-Level Size Zone Matrix (GLSZM), Neighboring Gray-Tone Difference Matrix (NGTDM), and transform-based features.

For intensity discretization, a bin width of 25 was used, and for wavelet transformation, 3 levels of decomposition were applied. These parameters were selected to ensure a balance between feature detail and computational feasibility.

To address overfitting, reduce feature redundancy, and enhance model performance, we implemented a multi-step feature selection process. Initially, we excluded features exhibiting a Pearson correlation coefficient with an absolute value ≥0.9 to mitigate multicollinearity, resulting in 290 remaining features. Subsequently, we employed the f_classif scoring function from the SelectKBest module of the scikit-learn library to assess the relevance of each feature. This function calculates the analysis of variance (ANOVA) F-values between the class labels in the training cohort and each radiomics feature, providing a measure of the linear correlation between features and target classes. To further refine the feature set, we applied the Least Absolute Shrinkage and Selection Operator (LASSO) algorithm. The final number of features selected by LASSO was determined by the optimal regularization parameter (lambda), which was identified through cross-validation. This step further isolated the most informative and predictive features for model training. To mitigate potential scanner-related variability, all radiomics features were Z-score normalized using the mean and standard deviation calculated from the training cohort. All subsequent feature selection and model development steps were performed using a nested cross-validation scheme to avoid information leakage.

### 2.6 Development and evaluation of the radiomics model

The radiomics model was developed through 10-fold cross-validation using various machine learning algorithms, including Logistic Regression (LR), Random Forest (RF), Support Vector Machine (SVM), Decision Trees (DT), K-Nearest Neighbors (K-NN), and Extreme Gradient Boosting (XGBoost). Prior to model training, all radiomics features were standardized using Z-score normalization (mean=0, variance=1) based on the parameters calculated from the training cohort.

To optimize performance, hyperparameter tuning was conducted for all classifiers using grid search within the training cohort. The parameter ranges tested were as follows: LR (penalty = 'l2'; C = [0.1, 1, 10, 100]), RF (n_estimators = [100, 200, 300]; max_depth = [None, 10, 20, 30]), SVM (C = [0.1, 1, 10, 100]; gamma = ['scale', 0.01, 0.001, 0.0001]; kernel = ['linear', 'rbf']), DT (max_depth = [None, 10, 20]; min_samples_split = [2, 5, 10]), K-NN (n_neighbors = [3, 5, 7, 9]), and XGBoost (n_estimators = [100, 200]; learning_rate = [0.01, 0.1]; max_depth = [3, 5, 7]).

Model generalizability was further assessed using an external validation cohort. Performance metrics included AUC, sensitivity (SEN), specificity (SPE), accuracy (ACC), positive predictive value (PPV), and negative predictive value (NPV). Throughout the model development and evaluation, the Elevated Risk group was defined as the positive class (coded as 1). The algorithm that achieved the highest overall performance was selected for constructing the final radiomics model.

**Figure 1:**
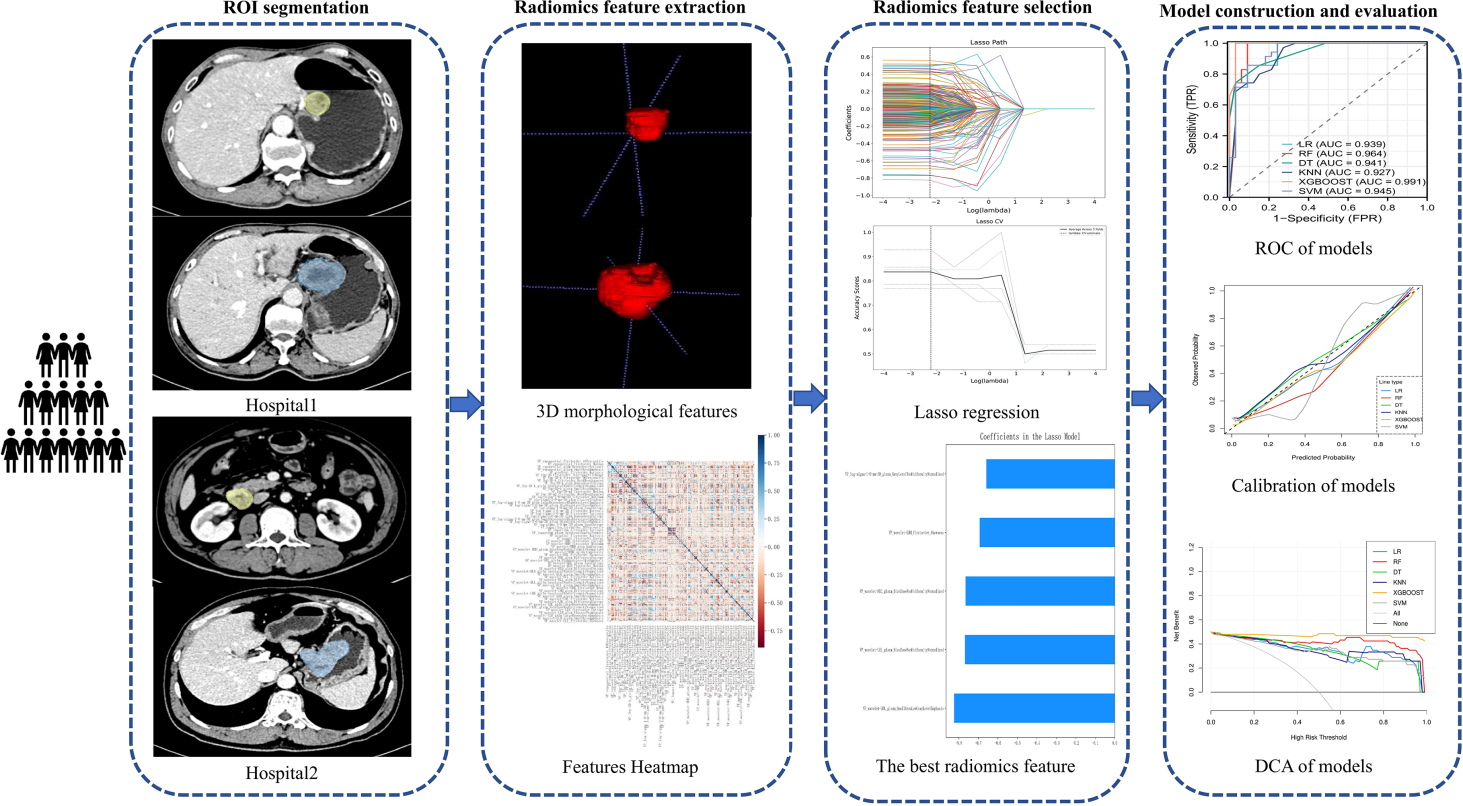
Workflow of this study.

To assess model calibration, calibration curves were constructed to evaluate the agreement between the observed outcomes and the predicted probabilities. Relying solely on statistical metrics is inadequate when assessing potential improvements to a risk model for clinical decision-making. Therefore, Decision Curve Analysis (DCA), a theoretical method for comparing the clinical performance of predictive models, was employed to gauge the models’ clinical utility. In DCA, the “treat-all” and “treat-none” strategies serve as reference points, representing two extreme scenarios. The further the decision curve deviates from these extremes, the greater the net clinical benefit provided by the model. The workflow of this study is shown in **Figure 1**.

### 2.7 Statistical analysis

Appropriate statistical tests were conducted to evaluate the significance of the findings. Pearson’s chi-square test was applied for categorical variables, while Student’s t-test or the Wilcoxon rank-sum test was used to compare continuous variables, depending on the distribution. Statistical significance was set at p < 0.05. All analyses were performed using SPSS (v24.0) and R (v4.1.0) software.

## 3 Results

### 3.1 Patient demographics and clinical features

Among the 123 patients with GISTs, the cohorts were divided into training (n=68; n=34 in the Lower Risk group), testing (n=30; n=17 in the Lower Risk group), and external validation (n=25; n=10 in the Lower Risk group) cohorts. Clinical features and radiomics parameters were comparable between the training and testing cohorts (p > 0.05), ensuring consistency across datasets for model development and evaluation (**Table 2**).

**Table 2:** Patient characteristics in the training and testing cohorts.

Characteristics	Training cohort	Testing cohort	P value
Samples, n (%)	68	30	
Age<60 years, n (%)	53 (54.1%)	18 (18.4%)	0.067
Gender, Female,n (%)	37 (37.8%)	16 (16.3%)	0.921
GI-bleed, n (%)	31 (31.6%)	15 (15.3%)	0.687
Abdominal pain, n (%)	27 (27.6%)	12 (12.2%)	0.978
Shape, n (%)			0.463
Complex	40 (40.8%)	20 (20.4%)	
Oval	28 (28.6%)	10 (10.2%)	
Boundary, n (%)			0.579
Obscure	39 (39.8%)	19 (19.4%)	
Clear	29 (29.6%)	11 (11.2%)	
Location, n (%)			0.488
Gastric	50 (51%)	20 (20.4%)	
Intestinal	18 (18.4%)	10 (10.2%)	
Cystic degeneration, n (%)	44 (44.9%)	18 (18.4%)	0.656
Diameter, median (IQR) (cm)	3 (2, 6)	4 (3, 6)	0.363
Calcification, n (%)	9 (9.2%)	7 (7.1%)	0.342
Rad-score, median (lQR)	0.53(-2.79,2.19)	0.97 (-0.92,2.57)	0.262

### 3.2 Predictive value of clinical and conventional imaging features

The multivariable logistic regression analysis revealed that none of the assessed clinical or conventional CT imaging features were independent predictors of the binary NIH risk stratification (Lower vs. Elevated Risk). The ORs for all variables approximated 1.0, with 95% CIs spanning null value (1.0), and p-values were all greater than 0.05. The detailed results are presented in **Supplementary Table S1**. Consequently, these variables were not incorporated into the final radiomics model.

### 3.3 Reproducibility of segmentation and radiomics features

The median Dice similarity coefficient was 0.91 (inter−quartile range: 0.86–0.94) for inter−observer agreement and 0.93 (0.89–0.96) for intra−observer agreement, indicating excellent spatial consistency of the manual segmentations.

For the five radiomics features selected in the final SVM model, inter−observer ICC values ranged from 0.85 to 0.93, and intra−observer ICC values ranged from 0.88 to 0.95 (**Supplementary Table S2**), demonstrating high feature−level reproducibility.

### 3.4 Radiomics model development and performance evaluation

After feature dimensionality reduction, five texture features (**Supplementary Table S3**) most strongly associated with GISTs risk stratification were identified. These features primarily quantify tumor heterogeneity (e.g., gray-level non-uniformity, zone size variability) and the asymmetry of intensity distribution, which are imaging correlates of underlying biological aggression. This 5-feature model was developed from a training cohort containing 34 Elevated Risk cases (events-per-variable ratio (EPV)= 6.8).

**Figure 2:**
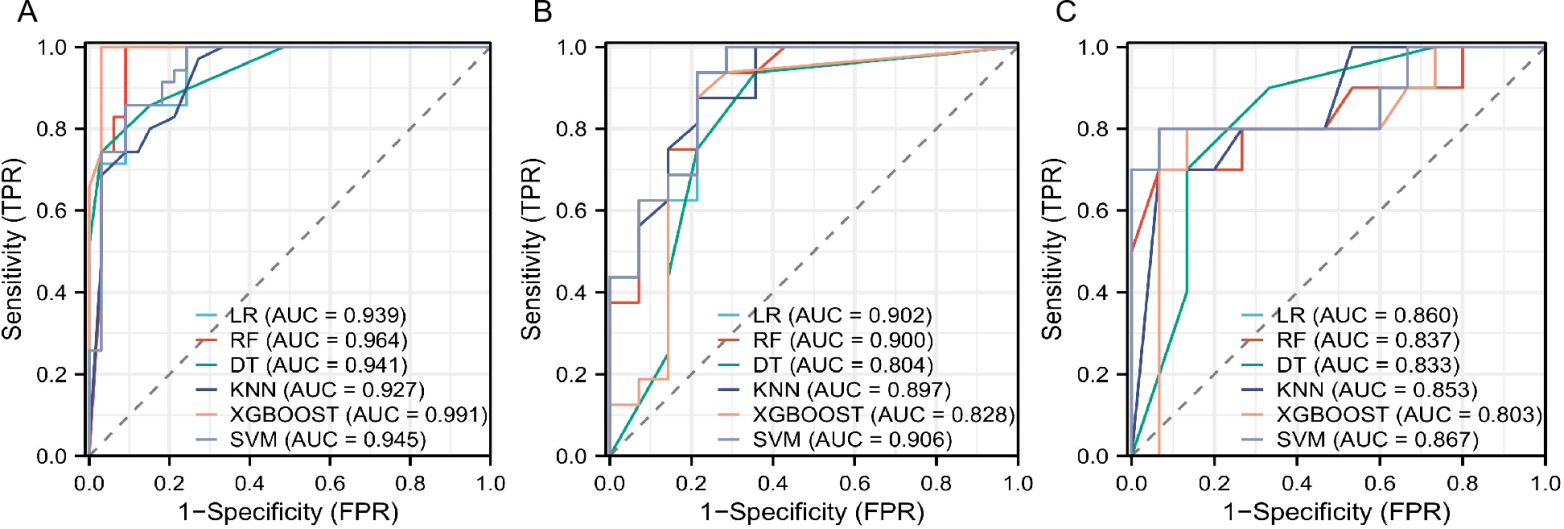
The ROC curves illustrate the predictive performance of six machine learning algorithms for distinguishing Elevated Risk from Lower Risk GISTs (Elevated Risk as the positive class). **(A)** Training cohort: The XGBoost model demonstrates the best performance with an AUC of 0.991, followed closely by SVM with an AUC of 0.945. **(B)** Testing cohort: SVM achieves the highest AUC of 0.906, highlighting its robust predictive capability. **(C)** External validation cohort: SVM outperforms other models with an AUC of 0.867, confirming its generalizability for GISTs risk stratification.

**Figure 3:**
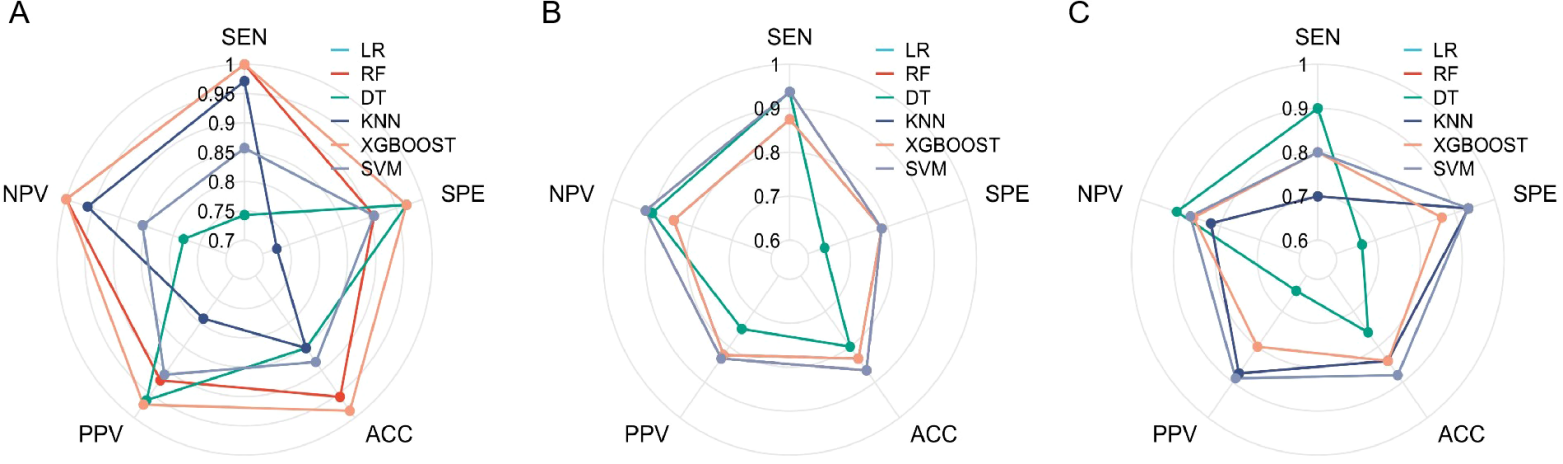
Radar charts comparing six machine learning algorithms based on performance metrics: SEN, SPE, ACC, PPV, and NPV. **(A)** Training cohort: XGBoost and RF exhibit the best overall performance. **(B)** Testing cohort: SVM demonstrates the most balanced performance across metrics. **(C)** External validation cohort: SVM outperforms other models, ensuring robust generalizability.

**Figure 4:**
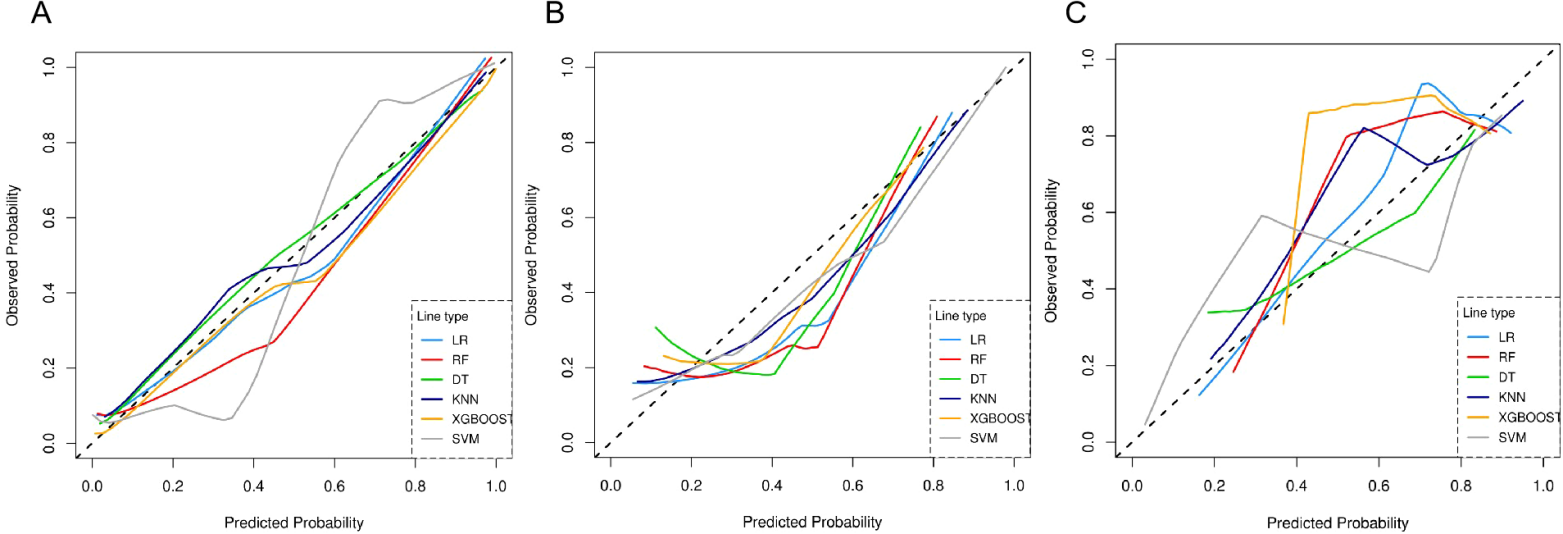
Calibration curves compare the predicted probabilities against observed outcomes for six machine learning models. The dashed diagonal line represents perfect calibration. **(A)** Training cohort. **(B)** Testing cohort. **(C)** External validation cohort.

**Figure 5:**
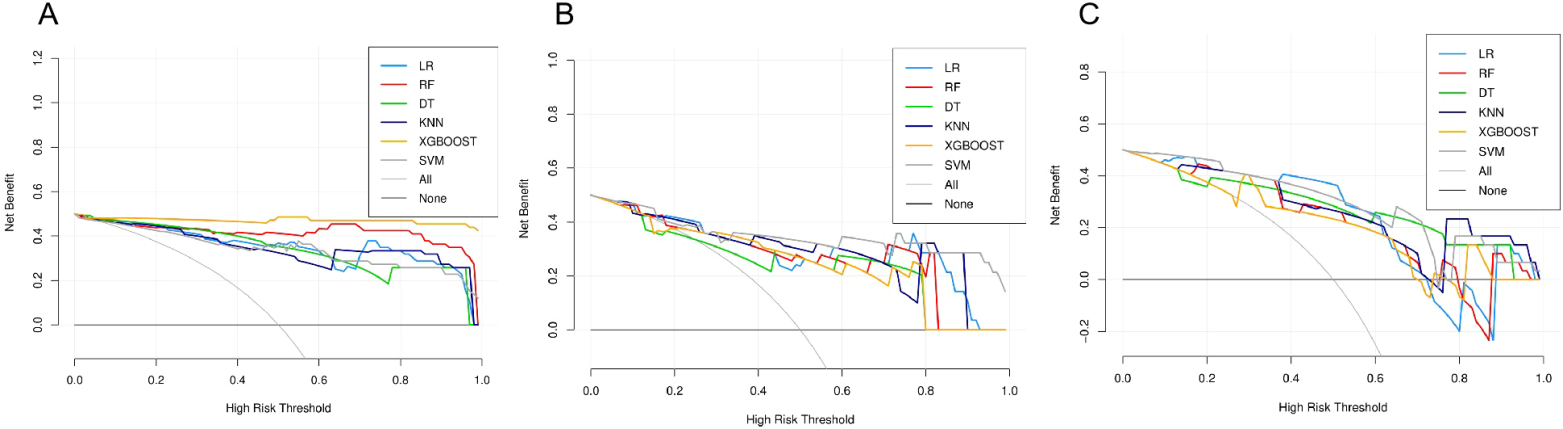
DCA curves compare the net clinical benefit of six machine learning models across different threshold probabilities for classifying Elevated Risk GISTs (Elevated Risk as the positive class). The “All” and “None” lines represent the extreme strategies of treating all or none of the cases, respectively. **(A)** Training cohort: XGBoost and RF demonstrate the highest net benefit across most threshold probabilities. **(B)** Testing cohort: SVM consistently provides superior clinical utility compared to other models. **(C)** External validation cohort: SVM achieves the best performance, confirming its ability to enhance clinical decision-making.

Using these features, a radiomics model was developed and evaluated using six different machine learning algorithms. All six algorithms demonstrated good predictive performance (**Figure 2**). Although the XGBoost classifier achieved the highest performance in the training cohort, the SVM classifier outperformed all others in the testing and external validation cohorts, achieving AUC values of 0.906 (95% CI: 0.812–0.964) and 0.867 (95%CI: 0.724–0.956), respectively. The optimal configuration—C=10, gamma=0.01, and an RBF kernel—was selected based on the highest AUC performance. Radar charts comparing the performance metrics (SEN, SPE, ACC, PPV, and NPV) of the six algorithms showed that RF and XGBoost achieved the best overall performance in the training cohort, while SVM exhibited the most balanced performance in the testing cohort and outperformed other models in the external validation cohort. For the final SVM model, a decision threshold of 0.5 was applied to the predicted probabilities for binary classification. On the external validation cohort, it achieved SEN = 0.800 (95% CI: 0.490–0.964), SPE = 0.933 (95% CI: 0.779–0.994), ACC = 0.880 (95% CI: 0.728–0.965), PPV = 0.889 (95% CI: 0.633–0.985), and NPV = 0.875 (95% CI: 0.655–0.970) ensuring robust generalizability (**Figure 3**). The corresponding confusion matrix for the external validation cohort is provided in **Supplementary Table S4**. Calibration curves demonstrated excellent agreement between the predicted and observed outcomes for all algorithms (**Figure 4**). Additionally, DCA revealed that the SVM algorithm provided higher net benefits across various threshold probabilities in the testing and external validation cohorts, highlighting its potential to enhance clinical decision-making (**Figure 5**). As a result, the radiomics model was finalized using the SVM algorithm, given its superior performance and clinical utility.

**Figure 6:**
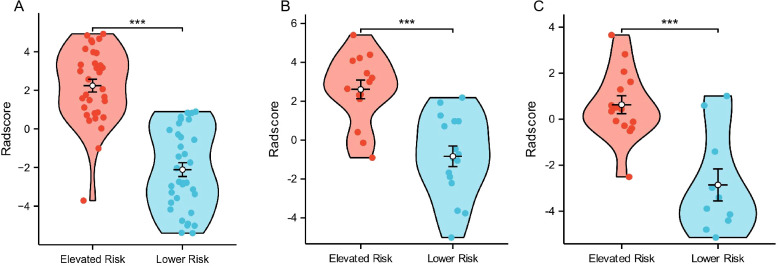
Violin plots display the distribution of Rad-scores between the Elevated Risk and Lower Risk groups in three cohorts. **(A)** Training cohort: The Elevated Risk group exhibits significantly higher Rad-scores compared to the Lower Risk group (p < 0.0001). **(B)** Testing cohort: A significant difference in Rad-scores is observed between the two groups (p < 0.001). **(C)** External validation cohort: Rad-scores are significantly higher in the Elevated Risk group (p < 0.0001). “***” indicates a statistically significant difference with p < 0.001.

The distribution of Rad-scores across all cases, based on the model formula (Equation 1), was visualized, revealing significantly higher scores in the Elevated Risk group compared to the Lower Risk group. This difference was statistically significant in the training cohort (p < 0.0001), testing cohort (p < 0.001), and external validation cohort (p < 0.0001), highlighting a robust association between higher Rad-scores and GISTs risk stratification (**Figure 6**).

Equation:

(1) $$\begin{array}{l} Rad\;Score\;=\;–0.6591\;\times \;VP\_log-sigma-1-0-mm-3D\_glszm\_GrayLevelNonUniformityNormalized\\ +\;0.7654\;\times \;VP\_wavelet-HHL\_glszm\_SizeZoneNonUniformityNormalized\\ +\;0.6929\;\times \;VP\_wavelet-LHH\_firstorder\_Skewness\\ +\;0.7685\;\times \;VP\_wavelet-LHL\_glszm\_SizeZoneNonUniformityNormalized\\ +\;0.8243\;\times \;VP\_wavelet-LHL\_glszm\_SmallAreaLowGrayLevelEmphasis \end{array}$$

## 4 Discussion

GISTs are associated with variable malignant potential and a significant risk of recurrence or metastasis, making accurate preoperative risk stratification essential for guiding treatment strategies (4). However, traditional methods, such as histopathological evaluation after surgery, are invasive and unable to provide preoperative guidance. Moreover, subjective imaging-based assessments often lack the precision to capture tumor heterogeneity and accurately predict risk levels (19). With advancements in artificial intelligence, machine learning-based radiomics has gained traction in medical imaging for extracting quantitative features that capture tumor characteristics (20–22). In this study, we focused on the application of CT-based radiomics combined with machine learning for the preoperative risk stratification of GISTs. Our findings demonstrated that the proposed model exhibited strong predictive performance.

In this study, the SVM classifier was selected to construct the radiomics model due to its optimal and balanced predictive performance. As shown in **Figure 2**, SVM achieved the highest AUC in both the testing (0.906) and external validation (0.867) cohorts. Its effectiveness in handling high-dimensional data and robustness against overfitting in our dataset are supported by previous literature (23, 24). Furthermore, the SVM model demonstrated superior calibration (**Figure 4**) and provided the greatest net clinical benefit across decision thresholds (**Figure 5**), confirming its reliability and potential clinical utility.

In addition, we explored whether the integration of clinical features—such as tumor size, anatomical location, and GI-bleed—could improve the performance of the radiomics model. However, multivariate logistic regression analysis indicated that none of these clinical variables served as independent predictors of risk stratification. Therefore, we did not incorporate them into the final model. This decision was made to maintain the model’s simplicity, reduce potential overfitting, and emphasize the predictive value of imaging-derived features.

The venous phase of contrast-enhanced CT imaging was selected for tumor delineation due to its superior visualization of vascularity and microvascular density, essential for GISTs risk stratification. Compared to arterial and delayed phases, the venous phase offers stable contrast enhancement, improving radiomics feature extraction (25–27). 3D-ROIs were utilized to capture the entire tumor volume, ensuring comprehensive analysis of tumor heterogeneity and reducing slice selection bias seen in 2D methods (28, 29). Although 3D annotation is more time-intensive, it provides richer data for predictive modeling. Experienced radiologists segmented images using the SHUKUN platform, enhancing data precision and the predictive model’s reliability.

Multi-class classification models have been explored in previous studies. For example, Chen et al. (30) and Mao et al. (13) achieved moderate success in distinguishing between low-risk, intermediate-risk, and high-risk GISTs. However, these multi-class models are often challenging to interpret in clinical settings, as the distinctions between intermediate and high-risk categories may not directly inform treatment strategies (17, 18). In contrast, although the current study by Rengo et al. (14) demonstrates promising performance in binary risk classification using combined CT features, it lacks independent external validation, which limits its generalizability. Our binary classification framework addresses this issue by focusing on the key clinical decision point—whether a patient requires adjuvant therapy. This simplification enhances the model’s utility in guiding preoperative decision-making.

This study provides several advancements over prior research. First, the external validation ensures that the model is applicable across different clinical settings and imaging protocols, addressing a common limitation of radiomics studies. Second, by integrating DCA, we demonstrated the model’s clinical utility across a range of threshold probabilities, highlighting its potential to optimize treatment decisions. Most importantly, the binary (Lower vs. Elevated Risk) output is designed for straightforward integration into clinical pathways. Specifically, a “Lower Risk” prediction could support a decision for surgical resection alone, while an “Elevated Risk” prediction would flag the patient for discussion of adjuvant therapy in a multidisciplinary team meeting, directly informing the key postoperative treatment decision.

While our study offers several strengths, certain limitations warrant further investigation: 1. The study’s sample size, reflected in a modest EPV ratio of 6.8 in the training cohort, remains a key limitation. This, coupled with the high initial feature dimensionality, underscores the potential for overfitting. We addressed this through rigorous internal validation and successful external testing. Nevertheless, future multi-center studies with larger cohorts are essential to improve model stability and provide stronger evidence for clinical application. 2. While this study did not introduce novel segmentation or feature extraction algorithms, our aim was to evaluate the clinical applicability of a standardized radiomics workflow using validated tools. Manual ROI segmentation was adopted to maximize anatomical accuracy, and features were extracted using the SHUKUN platform to ensure consistency and reproducibility. However, we acknowledge that this approach lacks methodological novelty. Future work may explore automated or deep learning–based segmentation and more advanced feature engineering techniques to further improve efficiency, reproducibility, and predictive performance. 3. This study focused exclusively on CT imaging. Integrating additional modalities, such as MRI or PET, could provide complementary information and improve predictive accuracy. 4. Combining radiomics features with genomic, proteomic, or metabolomic data may reveal deeper insights into GISTs biology and enhance the model’s prognostic power.

## 5 Conclusions

This study developed and externally validated a CT-based radiomics model for the preoperative risk stratification (Lower vs. Elevated Risk) of GISTs. The model demonstrated robust performance and generalizability. It may represent a useful step forward in addressing limitations of prior studies, including the lack of external validation. Our work provides preliminary evidence supporting the potential of radiomics to inform personalized treatment strategies, laying the foundation for more effective clinical decision-making. Future efforts should focus on expanding sample sizes, incorporating automated segmentation tools, and exploring multi-modal and multi-omics approaches to further enhance the utility and precision of radiomics-based risk stratification models.

## Data Availability

The original contributions presented in the study are included in the article/**Supplementary Material**. Further inquiries can be directed to the corresponding author.
