# Supplementary material for: A CT-based radiomics model for preoperative risk stratification of gastrointestinal stromal tumors
Source: Front Oncol. 2026 Feb 19;16:1671745. doi: 10.3389/fonc.2026.1671745 (PMC12960089; doi:10.3389/fonc.2026.1671745)
Supplement: Supplementary file 1 [file Table1.docx]

## ****TRIPOD-ML CHECKLIST****

****Title:**** A CT-Based Radiomics Model for Preoperative Risk Stratification of Gastrointestinal Stromal Tumors

****Corresponding Author:**** Wentao Dong

****Page numbers refer to the revised manuscript.****

### ****SECTION 1: TITLE AND ABSTRACT****

| **Item No.** | **Checklist Item** | **Reported on Page/Line** |
| --- | --- | --- |
| 1a | Identify the study as developing and/or validating a multivariable prediction model, the target population, and the outcome to be predicted. | Title, Abstract (Background, Objectives) |
| 1b | Provide a summary of objectives, study design, setting, participants, sample size, predictors, outcome, statistical analysis, results, and conclusions. | Abstract (Methods, Results, Conclusions) |
| 1c | State whether the study concerns the development, validation, or updating of a prediction model. | Abstract (“developed and validated”) |

### ****SECTION 2: INTRODUCTION****

| **Item No.** | **Checklist Item** | **Reported on Page/Line** |
| --- | --- | --- |
| 2a | Explain the medical context (including whether diagnostic or prognostic) and rationale for developing or validating the multivariable prediction model. | Introduction (Paragraph 1-2) |
| 2b | Specify the objectives, including whether the study describes the development or validation of the model, or both. | Introduction (Final paragraph) |

### ****SECTION 3: METHODS****

| **Item No.** | **Checklist Item** | **Reported on Page/Line** |
| --- | --- | --- |
| ****Source of data**** |  |  |
| 3a | Describe the study design or source of data (e.g., randomized trial, cohort, or registry data), separately for the development and validation data sets, if applicable. | Methods 2.1 (retrospective, multi-center) |
| 3b | Specify the key study dates, including start of accrual; end of accrual; and, if applicable, end of follow-up. | Methods 2.1 (Jan 2020 – Aug 2024) |
| ****Participants**** |  |  |
| 4a | Specify key elements of the study setting (e.g., primary care, secondary care, general population) including number and location of centres. | Methods 2.1 (two hospitals) |
| 4b | Describe eligibility criteria for participants. | Methods 2.1 (Inclusion/Exclusion criteria) |
| 4c | Give details of treatments received, if relevant. | N/A (Observational study, treatment not a predictor) |
| ****Outcome**** |  |  |
| 5a | Clearly define the outcome that is predicted by the prediction model, including how and when assessed. | Methods 2.1 (binary NIH risk: Lower vs. Elevated Risk, based on postoperative pathology) |
| 5b | Report any actions to blind assessment of the outcome to be predicted. | Outcome assessment is objective (pathology-based NIH criteria), not subject to blinding. |
| ****Predictors**** |  |  |
| 6a | Clearly define all predictors used in developing or validating the multivariable prediction model, including how and when they were measured. | Methods 2.4, 2.5 (Radiomics features from venous-phase CT) |
| 6b | Report any actions to blind assessment of predictors for the outcome and other predictors. | Methods 2.4 (Radiologists blinded to pathological risk outcome during segmentation) |
| ****Sample size**** |  |  |
| 7 | Explain how the study size was arrived at. | The sample size was determined by data availability during the study period |
| ****Missing data**** |  |  |
| 8 | Describe how missing data were handled (e.g., complete-case analysis, imputation) with details of any imputation method. | No missing data were present in the final included cohort |
| ****Statistical analysis methods**** |  |  |
| 9a | For development and validation of a prediction model, describe how predictors were handled in the analyses. | Methods 2.5, 2.6 (Feature selection: correlation filter, ANOVA, LASSO; ML algorithms) |
| 9b | Specify type of model, all model-building procedures (including any predictor selection), and method for internal validation. | Methods 2.5, 2.6 (SVM, hyperparameter tuning, 10-fold CV) |
| 9c | For validation, describe how the predictions were calculated. | Methods 2.6 (Model applied to external validation cohort) |
| 9d | Specify all measures used to assess model performance and, if relevant, to compare multiple models. | Methods 2.6 (AUC, SEN, SPE, ACC, PPV, NPV, calibration, DCA) |
| 9e | Describe any model updating (e.g., recalibration) arising from the validation, if done. | Not performed. |
| ****Risk groups**** |  |  |
| 10 | Provide details on how risk groups were created, if done. | Outcome is inherently binary (Lower vs. Elevated Risk). No post-hoc grouping of predicted probabilities. |
| ****Development vs. validation**** |  |  |
| 11 | For validation, identify any differences from the development data in setting, eligibility criteria, outcome, and predictors. | Methods 2.1 (Different hospital for external validation; same eligibility criteria, outcome, and imaging protocol). |

### ****SECTION 4: RESULTS****

| **Item No.** | **Checklist Item** | **Reported on Page/Line** |
| --- | --- | --- |
| ****Participants**** |  |  |
| 12a | Describe the flow of participants through the study, including the number of participants with and without the outcome and, if applicable, a summary of the follow-up time. A diagram may be helpful. | Figure 1 (Workflow of this study.) |
| 12b | Describe the characteristics of the participants (basic demographics, clinical features, predictors), including the number of participants with missing data for predictors and outcome. | Table 2, Table S1, Results 3.1, 3.2 |
| ****Model development**** |  |  |
| 13a | Specify the number of participants and outcome events in each analysis. | Results 3.1 (Training: 68, Testing: 30, External: 25; Elevated Risk cases: 34 in training) |
| 13b | If done, report the unadjusted association between each candidate predictor and outcome. | Results 3.2 (Multivariable analysis showed no independent predictors among clinical/imaging features). |
| ****Model specification**** |  |  |
| 14a | Present the full prediction model to allow predictions for individuals (i.e., all regression coefficients, and model intercept or baseline survival at a given time point). | Results 3.4 (Equation for Rad-score) |
| 14b | Explain how to use the prediction model. | Results 3.4 (Equation and threshold of 0.5) |
| ****Model performance**** |  |  |
| 15 | Report performance measures (with CIs) for the prediction model. | Results 3.4 (AUC, SEN, SPE, ACC, PPV, NPV with 95% CIs for testing and external cohorts) |
| ****Model-updating**** |  |  |
| 16 | If done, report the results from any model updating (i.e., model specification, model performance). | Not performed. |

### ****SECTION 5: DISCUSSION****

| **Item No.** | **Checklist Item** | **Reported on Page/Line** |
| --- | --- | --- |
| ****Limitations**** |  |  |
| 17 | Discuss any limitations of the study (such as nonrepresentative sample, few events per predictor, missing data). | Discussion (Limitations paragraph) |
| ****Interpretation**** |  |  |
| 18a | Give an overall interpretation of the results, considering objectives, limitations, and results from similar studies. | Discussion (Paragraphs 1-4) |
| 18b | Discuss the potential clinical use of the model and implications for future research. | Discussion (Final paragraph before limitations) |
| ****Other information**** |  |  |
| ****Funding**** |  |  |
| 19 | Give the source of funding and the role of the funders for the present study. | Declarations section |

### ****SECTION 6: SUPPLEMENTARY MATERIAL****

| **Item No.** | **Checklist Item** | **Status** |
| --- | --- | --- |
| N/A | The completed TRIPOD-ML checklist is provided as supplementary material. | Provided |
| N/A | Supplementary tables (S1-S4) are provided. | Referenced in text |
