# Supplementary material for: A CT-based radiomics model for preoperative risk stratification of gastrointestinal stromal tumors
Source: Front Oncol. 2026 Feb 19;16:1671745. doi: 10.3389/fonc.2026.1671745 (PMC12960089; doi:10.3389/fonc.2026.1671745)
Supplement: Supplementary file 2 [file DataSheet1.docx]

Table S1. Multivariable Logistic Regression Analysis of Clinical and Conventional Imaging Features for Predicting NIH Risk Stratification (Lower vs. Elevated Risk).

| **Variable** | **Category / Unit** | **Odds Ratio (OR)** | **95% Confidence Interval** | **P-value** |
| --- | --- | --- | --- | --- |
| ****Age**** | ≥60 vs. <60 years | 1.12 | 0.51 – 2.46 | 0.774 |
| ****Gender**** | Male vs. Female | 0.89 | 0.42 – 1.91 | 0.768 |
| ****GI-bleed**** | Yes vs. No | 1.35 | 0.64 – 2.83 | 0.426 |
| ****Abdominal pain**** | Yes vs. No | 1.08 | 0.52 – 2.26 | 0.834 |
| ****Tumor size**** | per 1 cm increase | 1.07 | 0.92 – 1.25 | 0.362 |
| ****Location**** | Intestinal vs. Gastric | 1.42 | 0.65 – 3.12 | 0.381 |
| ****Shape**** | Complex vs. Oval | 1.21 | 0.58 – 2.54 | 0.612 |
| ****Boundary**** | Obscure vs. Clear | 1.55 | 0.74 – 3.25 | 0.242 |
| ****Cystic degeneration**** | Yes vs. No | 0.94 | 0.45 – 1.96 | 0.868 |
| ****Calcification**** | Yes vs. No | 1.28 | 0.48 – 3.42 | 0.623 |

****Abbreviations:**** CI, confidence interval; GI-bleed, gastrointestinal bleeding.

Table S2. Inter- and intra-observer reproducibility of the five radiomics features retained in the final SVM model.

| **Radiomics Feature** | **Inter‑observer ICC (95% CI)** | **Intra‑observer ICC (95% CI)** | **Interpretation** |
| --- | --- | --- | --- |
| **VP_log-sigma-1-0-mm-3D_glszm_GrayLevelNonUniformityNormalized** | 0.89 (0.82–0.94) | 0.92 (0.86–0.96) | Excellent |
| **VP_wavelet-LHH_firstorder_Skewness** | 0.93 (0.88–0.96) | 0.95 (0.91–0.97) | Excellent |
| **VP_wavelet-HHL_glszm_SizeZoneNonUniformityNormalized** | 0.85 (0.77–0.91) | 0.88 (0.80–0.93) | Good to Excellent |
| **VP_wavelet-LHL_glszm_SizeZoneNonUniformityNormalized** | 0.87 (0.79–0.92) | 0.90 (0.83–0.94) | Excellent |
| **VP_wavelet-LHL_glszm_SmallAreaLowGrayLevelEmphasis** | 0.86 (0.78–0.91) | 0.89 (0.82–0.94) | Good to Excellent |

ICC, intraclass correlation coefficient; CI, confidence interval.

ICC interpretation: < 0.50 = poor; 0.50–0.75 = moderate; 0.75–0.90 = good; > 0.90 = excellent.

## **Table S3. Description of the five radiomics features retained in the final SVM model.**

| **No.** | **Feature Name** | **Category** | **Brief Interpretation** | **Coefficient in Rad‑score** |
| --- | --- | --- | --- | --- |
| 1 | VP_log‑sigma‑1‑0‑mm‑3D_glszm_GrayLevelNonUniformityNormalized | GLSZM (Log‑filtered) | Quantifies the heterogeneity of gray‑level intensities within the tumor; higher values indicate greater intra‑tumoral textural non‑uniformity. | –0.6591 |
| 2 | VP_wavelet‑HHL_glszm_SizeZoneNonUniformityNormalized | GLSZM (Wavelet‑HHL) | Measures the variability in the size of homogeneous texture regions in the high‑high‑low wavelet sub‑band; reflects spatial irregularity of tumor substructures. | +0.7654 |
| 3 | VP_wavelet‑LHH_firstorder_Skewness | First‑order (Wavelet‑LHH) | Describes the asymmetry of the voxel‑intensity distribution in the low‑high‑high wavelet sub‑band; positive skewness suggests a tail of higher intensity values. | +0.6929 |
| 4 | VP_wavelet‑LHL_glszm_SizeZoneNonUniformityNormalized | GLSZM (Wavelet‑LHL) | Similar to feature #2 but derived from the low‑high‑low wavelet sub‑band; captures multi‑scale texture inhomogeneity. | +0.7685 |
| 5 | VP_wavelet‑LHL_glszm_SmallAreaLowGrayLevelEmphasis | GLSZM (Wavelet‑LHL) | Emphasizes the presence of small, low‑attenuation (e.g., necrotic or cystic) regions within the tumor. | +0.8243 |

GLSZM, Gray‑Level Size Zone Matrix.

**Table S4. Confusion matrix for the SVM model on the external validation cohort (n=25) using a decision threshold of 0.5.**

|  | Predicted: Positive | Predicted: Negative | **Row Total** |
| --- | --- | --- | --- |
| **Actual: Positive** | 8 (True Positive, TP) | 2 (False Negative, FN) | **10** |
| **Actual: Negative** | 1 (False Positive, FP) | 14 (True Negative, TN) | **15** |
| **Column Total** | **9** | **16** | **25** |
